# Dynamic Changes in Microbiome Composition Following Mare’s Milk Intake for Prevention of Collateral Antibiotic Effect

**DOI:** 10.3389/fcimb.2021.622735

**Published:** 2021-04-21

**Authors:** Almagul Kushugulova, Ulrike Löber, Saniya Akpanova, Kairat Rysbekov, Samat Kozhakhmetov, Zhanagul Khassenbekova, Morgan Essex, Ayaulym Nurgozhina, Madiyar Nurgaziyev, Dmitriy Babenko, Lajos Markó, Sofia K. Forslund

**Affiliations:** ^1^ Laboratory of Human Microbiome and Longevity, Center for Life Sciences, National Laboratory Astana, Nazarbayev University, Nur-Sultan, Kazakhstan; ^2^ Kazakhstan Society of Human Microbiome Researchers, Nur-Sultan, Kazakhstan; ^3^ SaumalBioTech, Nur-Sultan, Kazakhstan; ^4^ Experimental and Clinical Research Center, a Cooperation of Charité-Universitätsmedizin Berlin and Max Delbrück Center for Molecular Medicine, Berlin, Germany; ^5^ Charité-Universitätsmedizin Berlin, Corporate Member of Freie Universität Berlin, Humboldt-Universität zu Berlin, and Berlin Institute of Health, Berlin, Germany; ^6^ Max Delbrück Center for Molecular Medicine in the Helmholtz Association, Berlin, Germany; ^7^ DZHK (German Centre for Cardiovascular Research), Partner Site Berlin, Berlin, Germany; ^8^ Department of Pediatric Diseases with Courses in Cardio-Rheumatology and Gastroenterology, Nur-sultan (Astana) Medical University, Nur-Sultan, Kazakhstan; ^9^ Laboratory of Innate Immunity, Department of Microbiology, Infectious Diseases and Immunology, Charité-Universitätsmedizin, Berlin, Germany; ^10^ Research Center Karaganda Medical University, Karagandy, Kazakhstan; ^11^ Structural and Computational Biology Unit, The European Molecular Biology Laboratory (EMBL), Heidelberg, Germany

**Keywords:** mare’s milk, microbiome, 16S rRNA gene sequencing, collateral antibiotic effect, intestinal immunity

## Abstract

**Introduction:**

Probiotics and prebiotics are widely used for recovery of the human gut microbiome after antibiotic treatment. High antibiotic usage is especially common in children with developing microbiome. We hypothesized that dry Mare’s milk, which is rich in biologically active substances without containing live bacteria, could be used as a prebiotic in promoting microbial diversity following antibiotic treatment in children. The present pilot study aims to determine the impacts of dry Mare’s milk on the diversity of gut bacterial communities when administered during antibiotic treatment and throughout the subsequent recovery phase.

**Methods:**

Six children aged 4 to 5 years and diagnosed with bilateral bronchopneumonia were prescribed cephalosporin antibiotics. During the 60 days of the study, three children consumed dry Mare’s milk whereas the other three did not. Fecal samples were collected daily during antibiotic therapy and every 5 days after antibiotic therapy. Total DNA was isolated and taxonomic composition of gut microbiota was analyzed by 16S rRNA amplicon sequencing. To assess the immune status of the gut, stool samples were analyzed by bead-based multiplex assays.

**Results:**

Mare’s milk treatment seems to prevent the bloom of Mollicutes, while preventing the loss of Coriobacteriales. Immunological analysis of the stool reveals an effect of Mare’s milk on local immune parameters under the present conditions.

## Introduction

Advances in studies of the human microbiome have shown that homeostasis of the intestinal microbiota is critical to maintaining health, especially in a developing organism. Antibiotics, which act to suppress pathogenic bacteria, and are usually prescribed for infections and infectious complications, also inevitably affect the normal microbiota. Antibiotics of various pharmacological groups have different effects on the composition and function of intestinal bacteria and lead to different consequences. It has been shown that changes in the intestinal microbiome in early childhood could play a role in the development of pathologies such as inflammatory bowel diseases, allergies, lactose intolerance, obesity, cardiometabolic disease, and even mental disorders including late dyslalia and autism (Adamek et al; Kushugulova et al., 2018a; Lee et al., 2018; Algazina et al., 2019; Pulikkan et al., 2019; Askarova et al., 2020)

It is known that destabilization of the microbiome composition, for example by antibiotics, results in the disruption of important microbiota functions, such as its trophic potential, its provision of short-chain fatty acids (SCFAs) to colonocytes, or its participation in bile acid metabolism (Taylor and Green, 2018; Parada Venegas et al., 2019). After an antibiotic course, it has been shown that bile acid and steroid metabolism are likely to decrease, while the metabolism of sugars and starch is likely to increase (Antunes et al., 2011). Destabilization leads to the disruption of interactions in the colonocyte-microbiota system, a decrease in the protective properties of the mucin layer and, as a consequence of these factors, a decrease in the colonization resistance of the microbiota with concomitantly increased risk for the growth of an opportunistic/pathogenic flora (Asha et al., 2006; Högenauer et al., 2006).

Antibiotic therapy has profound effect on the composition of the microbiome (Palleja et al., 2018). This can be especially deleterious in young children, where the destabilization of the microbiome can become a trigger factor in the development of chronic diseases. Antibiotics are believed to reduce colonization resistance against enterobacteria, such as *Escherichia coli* and *Salmonella enterica*, by increasing the inflammatory tone of the intestinal mucosa (Spees et al., 2013). Various studies have shown that different types of microbiome shifts can induce different types of immune cells, and different antibiotics can have different cytokine responses (Kosiewicz et al., 2011; Zheng et al., 2020). The mechanisms of these interactions are still relatively poorly defined in human studies.

In order to prevent damage to or restore the intestinal microbiome, probiotics and prebiotics are often used. Under certain circumstances use of probiotics could lead to severe complications such as endo cardiomyopathies or even septicemia (Lacaille et al., 2015; Naqvi et al., 2018; Suez et al., 2018) however, high intraindividual persistence alongside high interindividual variability in rapidly renewing microbiomes suggest that probiotics could be personalized based on knowledge of individual enterotype or other metrics of idiosyncrasy (Christensen et al., 2018; Costea et al., 2018; Yang et al., 2019; Wang et al., 2020) The wider use of functional foods or supplements that do not contain living microbial cells, such as prebiotics and metabiotics (Shenderov, 2013), may constitute a safer complementary practice to probiotic approaches, especially in vulnerable subjects, such as children.

Mare’s milk could be beneficial for recovering perturbed intestinal microbiome composition due to its prebiotic and immunomodulatory effects (Foekel et al., 2009; Wulijideligen Asahina et al., 2012; Suez et al., 2018) Antimicrobial activity of mare’s milk is associated with a high abundance of lysozymes, immunoglobulins, lactoperoxidases, and lactoferrin (Hancock et al., 2002; Pieszka et al., 2016; Kushugulova et al., 2018b). Additionally, it contains biologically active substances important for the intestinal microbiome and is close in biochemical composition to mother’s milk (Jastrzebska et al., 2017). Several studies have shown that breast milk feeding helps to recover the intestinal microbiota after intranatal and postnatal antibiotic therapy due to pre/probiotic and immunomodulatory effects, as well as to decrease the antibiotic resistance gene load (Azad et al., 2016; Pärnänen et al., 2018; Järvinen et al., 2019). We hypothesize that mare’s milk can help promote microbial diversity following antibiotic treatment. The present pilot study thus sought to determine the impacts of mare’s milk treatment on the diversity of the gut bacterial community when taken during antibiotic treatment and throughout the subsequent recovery phase, compared to antibiotic exposure in the absence of any other treatment.

## Materials and Methods

### Patients

This study was approved by the regional committee for medical research ethics in the National Laboratory Astana, Nazarbayev University (Protocol number 20 from 22.09.2017, IORG0006963), and registered on ClinicalTrials.gov under study identifier NCT03657836. Clinical investigations have been conducted according to the principles of the Declaration of Helsinki. Written informed consent for sample collection and subsequent analyses was obtained from the parents of all participants. Recruitment of patients was carried out in the Pulmonology Department of the First Children’s City Hospital, Nur-Sultan, Kazakhstan from September 2018 to August 2019.

**Figure 1:**
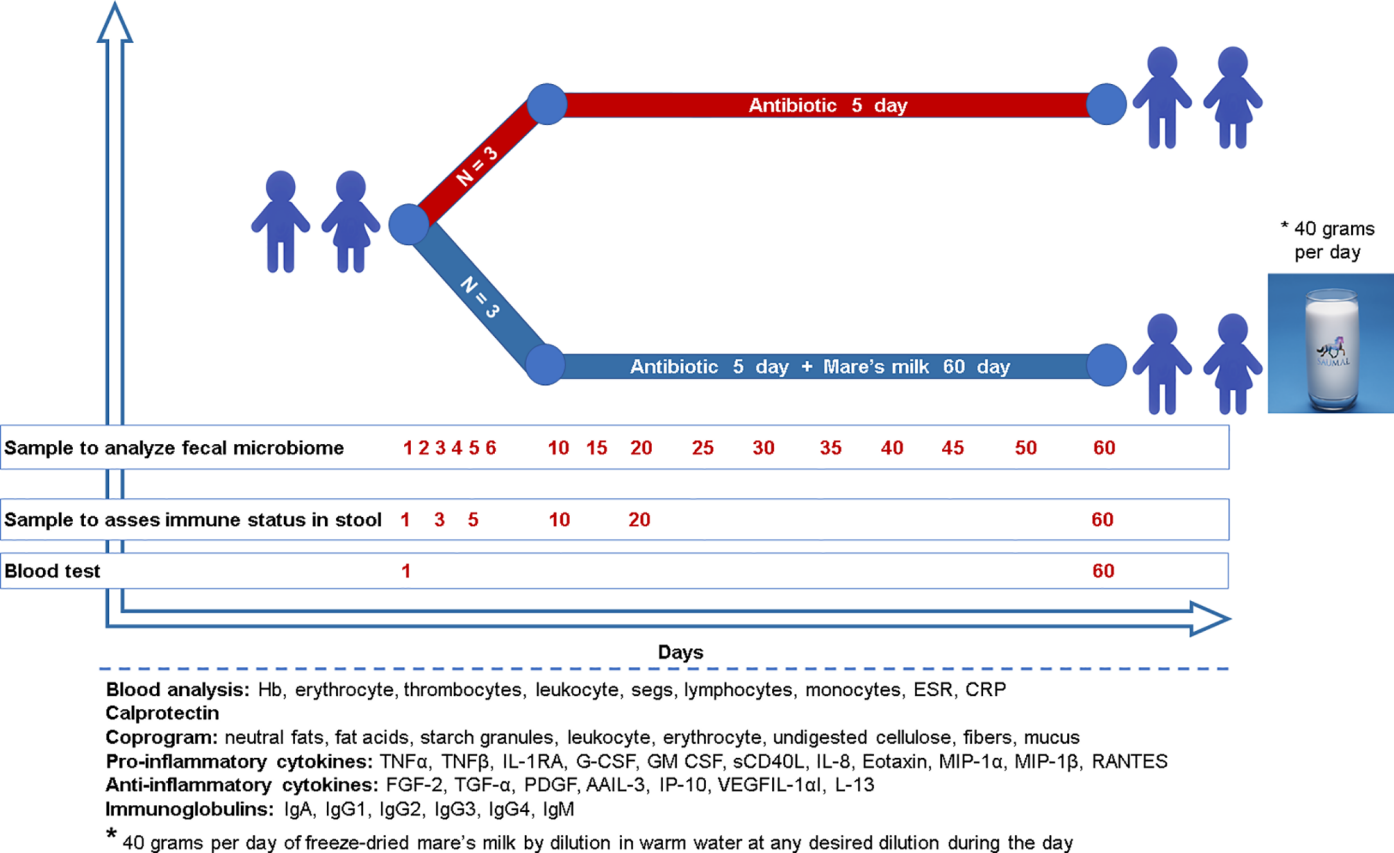
Study design Six children (age 3.9 ± 0.7) without previous antibiotic exposure were treated with a cephalosporin because of bronchopneumonia. Three children were randomly assigned to the intervention group and administered mare’s milk daily for the entirety of the 5-day antibiotic intervention as well as the 55 days following. Microbiome sampling (stool or swab) and other phenotyping were performed, as shown in the diagram for both study arms.

The study includes six children between the age of 4 to 5 years diagnosed with bilateral bronchopneumonia who met the inclusion and exclusion criteria. Inclusion criteria were patients of both sexes aged 4-5 years; the voluntary informed consent from the parents of the child to participate in the study; established diagnosis of acute upper respiratory tract disease with prescription of cephalosporin antibiotics; the duration of symptoms of acute upper respiratory tract disease no more than 72 hours before antibiotic treatment; and absence of prescription of antibacterial drugs during the last three months. Exclusion criteria were: a history of taking probiotics and antibiotics three months before admission to hospital; anamnesis of chronic diseases of the digestive tract and/or of any surgeries on the digestive tract; no history of concomitant diseases of the kidneys, liver, cardiovascular, chronic respiratory and other body systems, cancer, mental and decompensated endocrine diseases, tuberculosis, human immunodeficiency virus (HIV) infection; patient involvement in other clinical trials within the last three months; and finally lack of consent. The study lasted 60 days (**Figure 1**); patients were discharged on day seven and then observed on an outpatient basis. At the time of admission and on day 60 of the study, all patients were medically examined, which included a general blood test (hemoglobin (Hb), erythrocytes, thrombocytes, leukocytes, neutrophils, lymphocytes, monocytes, erythrocyte sedimentation rate (ESR), C-reactive protein (CRP)), stool test (coprogram), and fecal calprotectin quantification. During prescription of antibiotics of the cephalosporin group severity of the disease course and body weight were taken into account. Three out of six patients were additionally instructed to consume 40 grams per day of freeze-dried mare’s milk by dilution in warm water at any desired dilution during the day. Children were randomly assigned to a group which, along with standard therapy, received this auxiliary treatment in the form of reconstructed freeze-dried mare’s milk, which is encoded as MM (MM = mare’s milk) or to the control group, given antibiotics only (AB). In the control group, parents were instructed to refrain from giving their children any probiotics, prebiotics, or other functional products during the entire study period (60 days). Additionally, for 60 days during the collection of biomaterials, the investigator further advised parents to refrain from probiotics.

### Composition of the Mare’s Milk

The product is a freeze-dried powder made from fresh mare’s milk by vacuum drying. The product is manufactured at the mare’s milk factory of Eurasia invest Ltd LLP. The product was provided for the study participants in 20-gram sachets. Product expertise was conducted at the Kazakh Academy of Nutrition and SGS-CSTC Standards Technical Services Co., Ltd. (Shanghai). According to the results of the latter, sublimated mare’s milk contains the following micronutrients: all interchangeable and essential amino acids 17.18% (GB 5009.124-2016), saturated (3.54 g/100 g) and unsaturated fatty acids (5.24 g/100 g), ω-3 fatty acid (2.17 g/100 g) and ω-6 fatty acid (1.03 g/100 g), SCFA (0.02-2.13 g/100 g), vitamin B12 (0.77 μg/100 g), retinol (41 μg/100 g), α-Tocopherol (0.31 mg/100 g), vitamin E (0.31 mg/100 g), pantothenic acid (3.09×10^3^ μg/100 g), vitamin B6 (as pyridoxine) (0.155 mg/100 g), folic acid (10.1 μg/100 g), vitamin C (97 mg/100 g), zinc (Zn) (18.4 mg/kg), calcium (Ca) (6.66×10^3^ mg/kg), iron (Fe) (2.3 mg/kg), aspartic acid (1.46%), threonine (0.7%), serine (0.97%), glutamic acid (3.65%). Mare’s milk is further known to contain components important for the intestinal microbiome, such as lysozyme, lactoferrin, and lactadherin (Pieszka et al., 2016).

### Multiplex Immunoassay

To evaluate the immunological parameters, 3-4 mg of frozen feces of the samples were diluted in 200 μl of phosphate buffer, centrifuged, and the supernatant was analyzed using the manufacturer’s protocol. To analyze multiple cytokine and chemokine, we used the MILLIPLEX MAP Human Cytokine/Chemokine Magnetic Bead Panel, and for immunoglobulin isotyping the Milliplex^®^ magnetic bead panel (HGAMMAG-301K-06, EMD Millipore Corp., Billerica, MA) was used. Samples were analyzed on a Bio-Plex 3D suspension array system.

### Sample Collection and Data Analysis

Fecal samples were collected by the patients using swabs or tubes. Feces was deposited into a toilet hat and immediately sampled, then stored frozen at approximately −20°C in bags with a refrigerant. Biomaterial sampling was carried out by two methods because many children had constipation after antibiotic therapy. We used special bags with refrigerant inside which tubes were frozen, allowing us to transport them without violating the temperature regime. Samples were subsequently transferred to the laboratory and stored at −80°C until processed. Samples from patients were obtained prior to antibiotic therapy and between 1 to 60 days of the study, with daily collection until day 6, and thereafter collection every five days until day 60.

DNA was extracted from fecal samples using the Zymo Research Quick-DNA™ Fecal/Soil Microbe. Microprep Kit (Cat.No.: D6012), from swab samples using ZymoBiomics DNA Microprep kit (Cat No.: D4301), according to the manufacturer’s instructions. Fecal DNA concentrations were measured using a Nanodrop 2000/2000с (ThermoFisher).

Sequencing libraries were prepared by amplifying the V1-V3 region of the 16S rRNA gene according to the manufacturer’s instructions. The libraries’ quality was quantified on a Qubit 2.0 instrument using the dsDNA BR Qubit assay kit (ThermoFisher, 32853). Sequencing was performed as previously described (Su et al., 2018) on an Illumina MiSeq platform using 2 × 150 bp paired-end reads.

### Statistics

We performed data analysis using the less operational taxonomic units scripts (LotuS) pipeline (Hildebrand et al., 2014). The LotuS pipeline performs quality controls, sequence trimming and filtering, clustering OTUs, and taxonomic annotation from amplicons of metagenomic samples. LotuS was used with the sdm MiSeq configuration for sequence preprocessing. Taxonomic assignment of OTUs was performed using the databases SILVA (v138), greengenes (v13-5) and HITdb (v1.00) incrementally. Results were normalized using the rarefaction toolkit (RTK) pipeline with default parameters (Saary et al., 2017), to the lowest read count of the samples included following quality control. As a measure of alpha diversity, the Shannon index was calculated by RTK. Data was reformatted for analysis using the R statistical programming environment by means of custom Perl scripts. Beta diversity was assessed using the R package vegan (Bates et al., 2015; Oksanen et al., 2019), including PERMANOVA analysis and generation of distance metrics. Further analysis of taxonomic and immune cell data was conducted using linear mixed models compared using likelihood ratio tests as implemented in R packages lme4 and lmtest (Zeileis and Hothorn, 2002; Bates et al., 2015), using the lrtest function and ensuring REML approximation was not used Plots were generated using the ggplot2 R package (Hadley Wickham, 2016). For each analyzed variable, the Cliff’s delta effect size was evaluated by using the R package “orddom” (Rogmann, 2013). For results of the multiplex assay, statistical analysis was carried out using the R psych package (William Revelle, 2017) and standard t-tests. FDR adjustment was done using the Benjamini-Hochberg method.

## Results

At the time of discharge from the clinic, all patients exhibited a positive clinical response to antibiotic treatment, as assessed by qualitative amelioration of appetite and sleep, and normalization of body temperature. At baseline there were no significant differences in most of the clinical parameters (p < 0.17, p < 0.08, p < 0.45, and p < 0.51 for age, ESR, CRP, and fecal calprotectin, respectively), though white blood cell count was higher in the MM group as compared to the AB group (p < 0.01). By the 60th day of the study, all blood parameters in both groups were restored to normal and were not different between the two groups (**Table 1**).

**Table 1:** Baseline.

Name of indicators	AB		MM	
	baseline	60 day	baseline	60 day
	n=3	n=3	n=3	n=3
**Age**	4.4	4.4	3.5	3.5
**White Blood Cell, ^10^9^/L**	10.4 ± 1.21	8.0 ± 0.9	19,1 ± 1.24	8.9 ± 3.9
**ESR, mm/h**	9.33 ± 3.48	8.3 ± 6.8	20.6 ± 3.33	8.0 ± 6.2
**CRP, mg/L**	7.28 ± 4.73	1.8 ± 1.9	30.74 ± 24.74	1.45 ± 0.35
**Fecal Calprotectin, μg/g**	51.3 ± 16.2	19.1 ± 14.9	30.02 ± 23.76	25.6 ± 35.8

As expected, cephalosporin treatment led to a profound gut bacterial depletion. We observe an altered taxonomic composition during treatment even though no significant impact on alpha diversity (Shannon index) could be demonstrated using the present dataset (**Figure S1**), which is likely a statistical power limitation due to the small sample size. Similarly, while trends in shifting abundance are seen in various taxa - many persisting by the end of the study period - these changes do not achieve significance in the present pilot.

**Figure 2:**
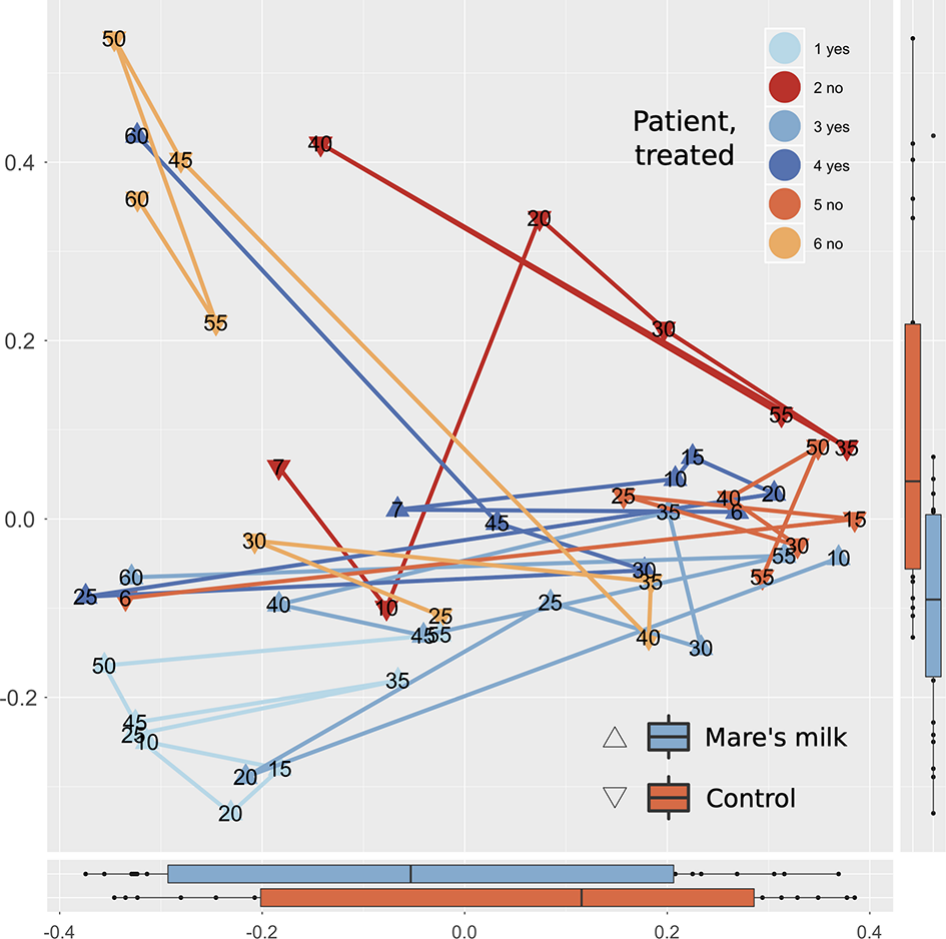
Multivariate analysis of gut composition shifts. Principal Coordinates Analysis (PCoA) of gut composition (samples post-antibiotic only, replicates not shown) analyzed using Bray-Curtis distances applied to rarefied gut 16S OTU data. The first two dimensions capturing the data distance space significantly (PERMANOVA P < 0.001, with parameters in order: intervention, sample ID, swab vs. stool sample, and time point) separates mare’s milk-treated samples from controls. Marginal plots show this distribution on both axes, and lines connect labeled time points of samples from the same subject. On the overall level, gut composition post-antibiotics differs between controls and MM-treated subjects.

PERMANOVA analysis of beta diversity (Bray-Curtis intersample dissimilarities) revealed a significant difference between samples from the AB and MM groups (**Figure 2**), including all time points following the cessation of antibiotic therapy.

**Figure 3:**
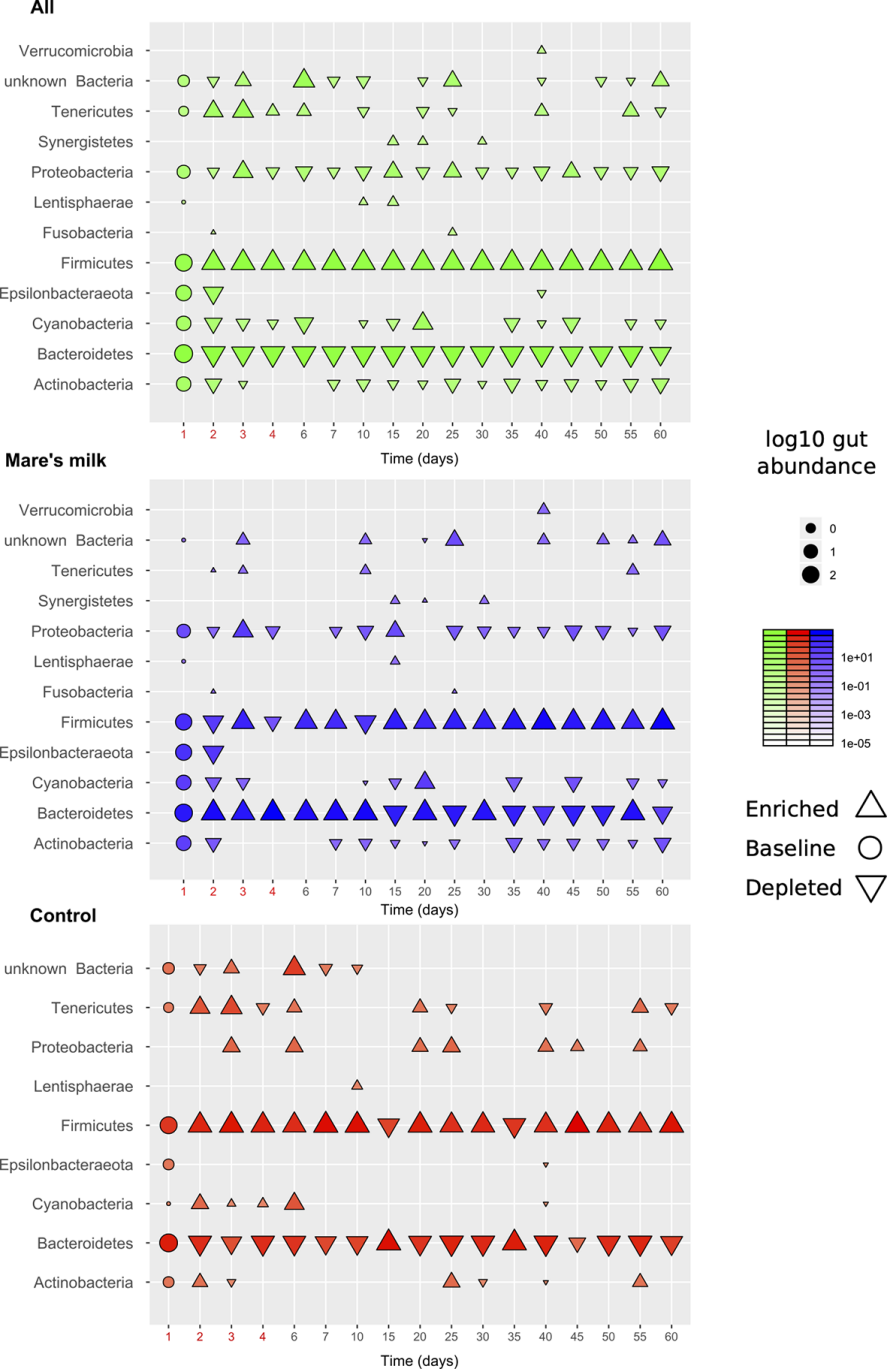
Overview of gut composition shifts at phylum level. The diagram shows gut abundance of different bacterial phyla, on average across the cohort (N=6, top panel), the intervention samples (N=3, middle panel), and the control samples (N=3, bottom panel). Marker size and intensity show average gut abundances with direction (upwards-facing: enriched, downwards-facing: depleted) compared to baseline (round markers). Most trends under the intervention (antibiotics: first 5 days; mare’s milk: all 60 days) are seen in both groups but with exceptions. Various taxa are gained or lost below detection threshold during the observation period.

While the small sample size limits our statistical power, clear trends are observable throughout the full length of the study. This allows us to assess the influence of the intervention on the gut abundance of specific taxa (**Figure 3**). Compositional bacterial microbiome analysis showed that antibiotic therapy positively correlates with the phylum Firmicutes (| δ | = 0.49; FDR < 0.30), mostly due to the order Clostridiales (| δ | = 0.43; p < 0.05; FDR < 0.39) and negatively correlated with Bacteroidetes (| δ | = -0.39; FDR < 0.41). The abundance of Erysipelotrichia decreases after exposure to antibiotics (| δ | = -0.48; p < 0.02; FDR < 0.43) and is still reduced by the end of the study period of 50-60 days (| δ | = -0.37; FDR < 0.33). The abundance of Deltaproteobacteria increases at the background of antibiotic therapy compared with baseline (| δ | = 0.35; FDR<0.36) and continues to trend until the end of the study period; effect size comparing baseline 50-60 days was | δ | = 0.54 (FDR<0.35). At the genus level, the abundance of *Hungatella* (| δ | = ‑0.28; p < 0.02; FDR < 0.44) and *Lachnoclostridium* (| δ | = -0.36; p < 0.03; FDR < 0.91) tends to decrease. We observe a trending negative correlation between antibiotic use and the abundance of an unclassified *Bacteroides* (| δ | = -0.38; p < 0.005; FDR < 0.67), genera of family Gastranaerophilales (| δ | = 0.28; p < 0.051; FDR < 0.67), and a positive correlation for *Escherichia-Shigella* (| δ | = -0.27; p < 0.04; FDR < 0.67).

**Figure 4:**
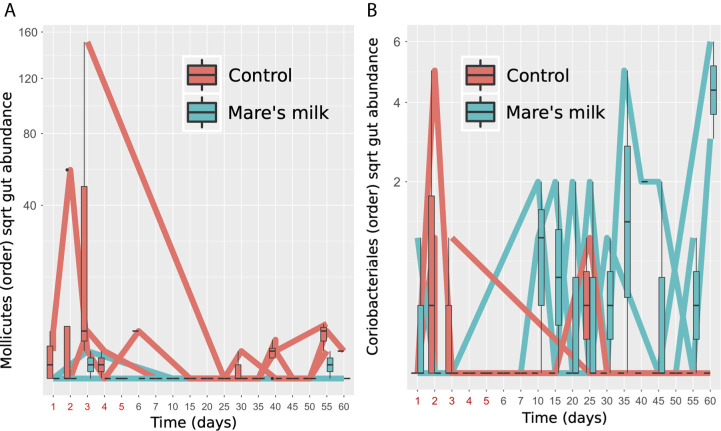
Specific gut orders enriched and depleted. Two bacterial orders show significantly altered abundance between MM-treated subjects and controls in post-antibiotic samples (replicates removed) tested using nested mixed models compared using a likelihood ratio test, controlling for donor as random effect, timepoint and stool vs. swab as fixed effects. **(A)** Figure shows Mollicutes gut abundance of the subjects, labeled red (N=3, controls) or blue (N=3, MM), shown as time trajectories and boxplots. **(B)** Figure shows Coriobacteriales gut abundance of the subjects, labeled red (N=3, controls) or blue (N=3, MM), shown as time trajectories and boxplots.

During the entire study period in group AB, the abundance of *Lachnospiraceae UCG-004*, *Phascolarctobacterium*, *Ruminococcaceae UCG-004*, *Ruminococcaceae UCG-005*, and *Fusicatenibacter* is higher than at the baseline for genera (**Table S1**). Despite limited statistical power in our pilot sample, analysis of gut taxonomic composition on the class level revealed significant (Benjamini-Hochberg FDR corrected p-values Q < 0.1) differences in the post-antibiotic trajectories of MM and AB samples, under mixed-effect model comparisons taking individual variability, time point, and stool/swab sample status into account. Mare’s milk treatment appears here to prevent the bloom of *Mollicutes* while preventing the loss of Coriobacteriales (**Figure 4**).

In the MM group, we instead observe a significant increase of the abundance of Coriobacteriales, which are in the phylum of Actinobacteria and consist of two families, the Coriobacteriaceae and Atopobiaceae. Coriobacteriales belong to the commensal flora and typically inhabit the oral cavity, gastrointestinal tract, and genital tract. In the intestines, the representatives of Coriobacteriales order are the Coriobacteriaceae family, which performs important functions, such as the conversion of bile salts and steroids, as well as the activation of food polyphenols (Clavel et al., 2014).

To determine trends in local intestinal immunity, we calculated the Cliff’s delta effect sizes for cytokines/chemokines comparing baseline with days 3, 5, 10, 20, and 60 of the study (**Table S2**).

On day 3 of the study, we observe the level of IgG3 lower than at the baseline (| δ | = -1.0; p < 0.03; FDR < 0.66). On day 10 of the study the decreased level of Interleukin 8 (IL8) (| δ | = -1.0; p < 0.03; FDR < 0.34) and Granulocyte-Colony Stimulating Factor (G-CSF) (| δ | = -1.0; p < 0.03; FDR < 0.34).

Antibiotic therapy led to significant decreases of anti-inflammatory cytokine/chemokine levels.

The analysis of absolute numbers of the pro-inflammatory and inflammatory cytokines/chemokines already on the 3rd day of the antibiotic therapy showed the changes in Macrophage Inflammatory Proteins αalpha (MIP1α) (FDR < 0.01), Tumor Necrosis Factor alpha (TNFα) (FDR < 0.02), GCSF (FDR < 0.09), IL1RA (FDR < 0.09) (**Table S3**). Transforming Growth Factor Alpha (TGFα) in healthy subjects strengthens proliferation of enterocytes and induces an intestinal adaptation. Here we find that after antibiotic therapy, the TGFα is decreased significantly by the 20th day of the study (FDR < 0.003) and remains low compared with the data before the start of antibiotic therapy until the 60th day of the study (FDR < 0.001). By the end of the study, we observe a decrease of soluble Cluster of Differentiation 40 ligand (sCD40L) level (FDR < 0.03).

During the entire period observed after antibiotic therapy, a significant decrease in the level of immunoglobulins is noted (**Table S4**); on day 3 in the AB group, levels of IgA (FDR < 0.07), IgG3 (FDR < 0.05), and IgM (FDR < 0.15) were significantly reduced. The trend persists throughout the observation period.

**Figure 5:**
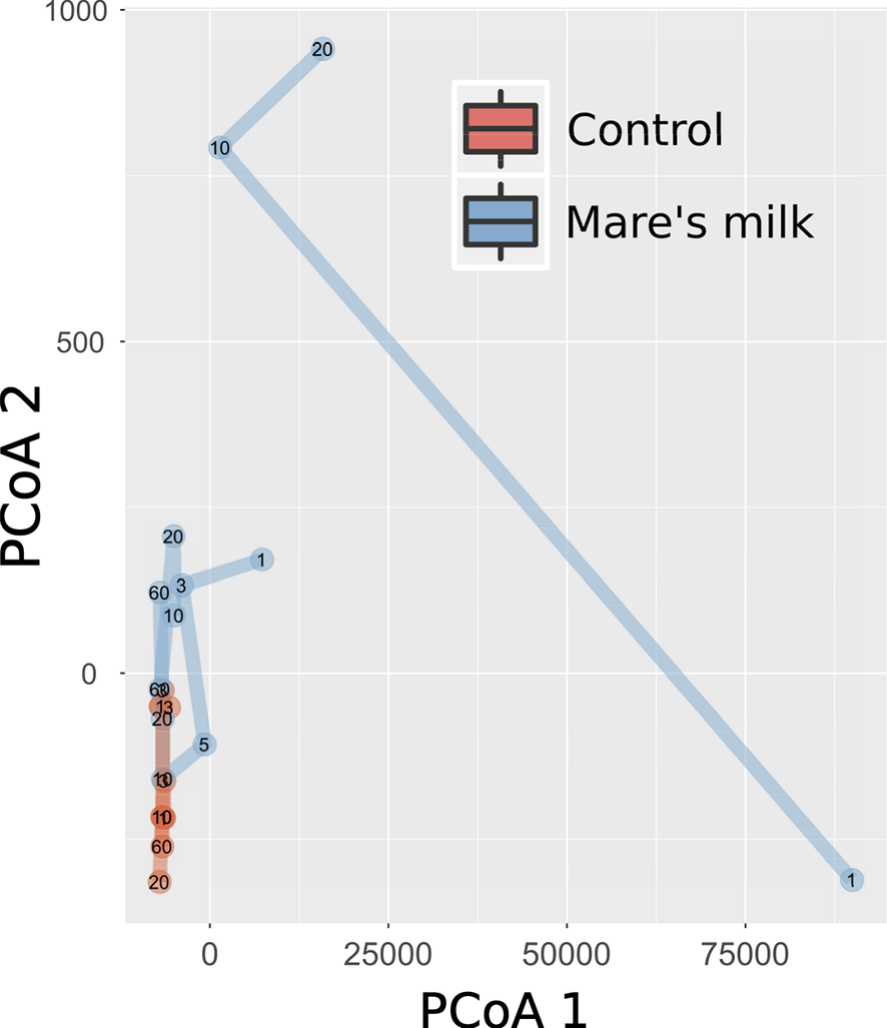
Multivariate analysis of immune shifts. Principal Coordinates Analysis (PCoA) of gut composition (samples post-antibiotic only, replicates not shown) analyzed using Bray-Curtis distances applied to immune measurements. The first two dimensions capturing the data distance space significantly (PERMANOVA P < 0.001, with parameters in order: intervention, participant identity, and time point) separates mare’s milk-treated samples from controls.

In the group receiving mare’s milk, we observe a significant change in Vascular Endothelial Growth Factor (VEGF) during all study periods (p < 0.06, FDR < 0.10). Some indicators significantly change by the 10th day of the study, IL3 (p < 0.03, FDR < 0.11), IL8 (p < 0.002, FDR < 0.005), TNFα (p < 0.02, FDR < 0.07), GCSF (p < 0.03, FDR < 0.11), which indicates an active inflammatory process in the body. However, in the following days, no significant changes were observed. Only the level of immunoglobulin G3 is reduced during the entire study period (p < 0.04, FDR < 0.08). Thus, taken together, complex immunological analysis reveals an effect of mare’s milk on local immune parameters under the present conditions (**Figure 5**).

As native gut inhabitants, opportunistic flora does not typically cause complications in a healthy host. However, immunosuppression may allow these bacteria to proliferate at a relative advantage in the intestinal tract and cause disseminated infections. In response to antibiotic therapy, we do observe changes in immunological parameters. However, it should be taken into account that at the time of the first measurements of the immune status, all patients had an inflammatory process as reflected in their blood counts on the first day of the study. To further elucidate, it will be necessary to expand this research and include the study of local intestinal immunity among a healthy group with and without mare’s milk consumption.

## Discussion

The human gut microbiome has coevolved with its host and is shaped by its diet and immune status, as well as by local competition of bacterial cells for an ecological niche and access to nutrients. A perturbed microbiome has a decreased capacity to perform its vital functions, such as digestive and synthetic activity, as well as providing colonization resistance and to regulate the body’s immune system. Antibiotic treatment has been shown to have major impact on the overall taxonomic composition of gut microbiota. Here, we studied the potential protective properties of mare’s milk on ameliorating adverse effects of antibiotic therapy on the gut microbiome.

**Figure 6:**
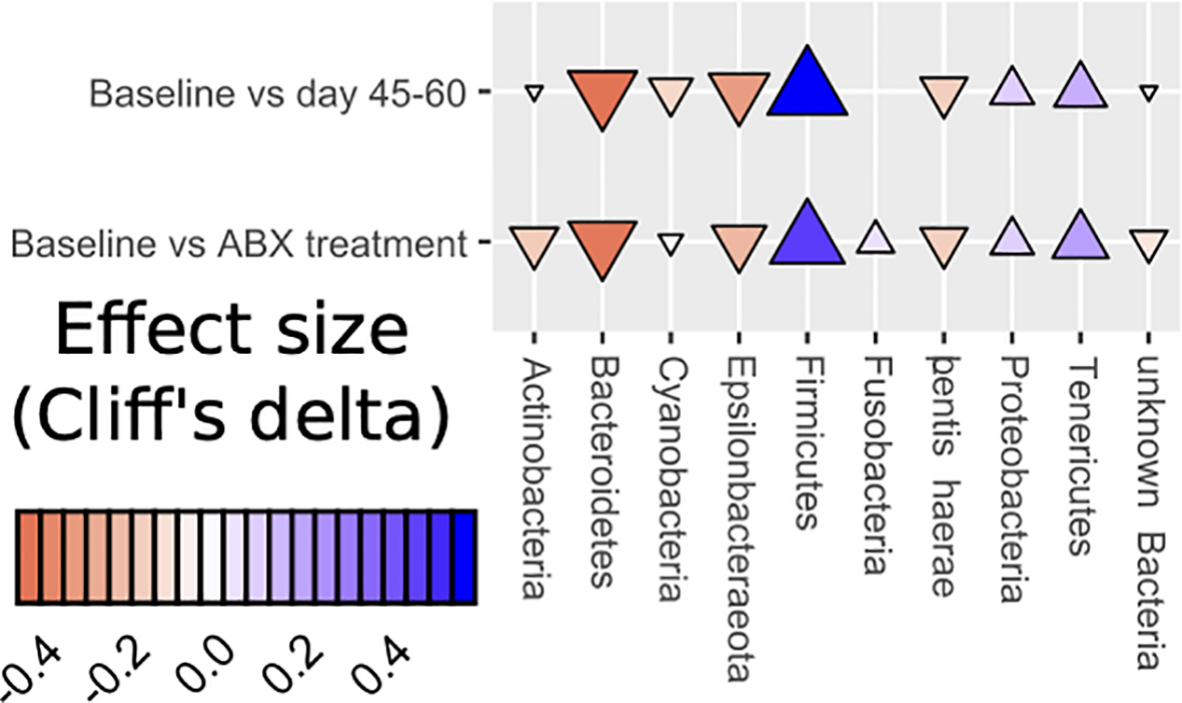
Dynamics of changes in abundance at the phylum level (Effect size Cliff’s delta).

We observe a perturbed microbiota following a short-term administration of cephalosporins. The effect of the antibiotic on the intestinal microbiota was pervasive and quick, with a loss of diversity and a change in the composition of the community seen only two days after the introduction of cephalosporins (Bhalodi et al., 2019). While the communities began to return to their original state by the 10th day of the study, this return was not fully accomplished, in line with other recent work (Palleja et al., 2018; Suez et al., 2018). In this study, the intestinal microbiota before antibiotic treatment consisted mainly of bacteria from the phyla Firmicutes, Bacteroidetes, Actinobacteria, Proteobacteria, and Epsilonbacteraeota. Post-antibiotics, fecal samples from the AB group exhibited an enrichment of Firmicutes and Tenericutes (**Figure 6**).

Further taxonomic characterization revealed the prevention of growth of Mollicutes in the MM group. The class Mollicutes contains more than forty known pathogens in humans and animals. Since Mollicutes class representatives lack cell walls, the main classes of antimicrobial agents, such as beta-lactam antibiotics, glycopeptides, and fosfomycin, do not affect them  (Chernova et al., 2016), this may explain the relatively high growth of these organisms in our studies as they would possess a relative advantage. Furthermore, beta-lactam antibiotics like the cephalosporins tested here are immunosuppressive, with the resulting inflammatory process potentially caused by representatives of the Mollicutes class (Huegin et al., 1986; Villa et al., 1991). Some Mollicutes cause human and animal diseases characterized by lymphoid infiltrates, immunosuppression, autoantibody production, and they have been associated with obesity (Turnbaugh et al., 2008). We propose that the inhibition effect we here see on Mollicutes under mare’s milk intervention may be due to lysozyme and lactoferrin acting as antimicrobial molecules, as well as through stimulating growth of commensal microbiota including the Coriobacteriales. Investigating the potential of dietary interventions to reduce collateral antibiotic effects on the microbiome is a growing area of research. Our exploratory study is one of the first microbiome analyses assessing the influence of mare’s milk. It was previously reported (Foekel et al., 2009; Pieszka et al., 2015; Shariatikia et al., 2017) that mare’s milk has beneficial effects on human health, and we proposed that its rich content of biologically active substances are beneficial for microbiome inhabitants. Our present preliminary results suggest that mare’s milk supplementation is associated with an improvement in post-antibiotic microbiome state relative to no intervention.

A significant difference in the trajectories of the MM and AB groups was nevertheless visible both in multivariate and univariate analyses of microbiome composition, including enrichment of Coriobacteriales bacteria. Members of this order are typically sensitive to antibiotic exposure, and apparently, mare’s milk contains substances able to support their growth.

Bacterial components of the gut microbiome have been previously shown to interact with the mucosal and systemic immune systems through specific pathways and cytokines. The mechanisms underlying the microbiome-cytokine interaction may involve mediation by the metabolites originating in the gut (Schirmer et al., 2016). It has further previously been observed that antibiotic-induced perturbations of the microbiome can modulate the inflammatory state of the host. To understand how antibiotics affect immune status, previous researchers analyzed effects of monoantibiotics or mixtures of broad-spectrum antibiotics (Sun et al., 2019) in experimental studies in various laboratory animal models, as well as monoantibiotics specific for gram-positive (Strzepa et al., 2017) or gram-negative bacteria (Shi et al., 2018). Thus, these previous studies offer potential scenarios for how microbiome-immune system interplay may be salient under the conditions we test here, both with regards to antibiotics administration and to possible partial mitigation of dysbiosis from the MM intervention.

Antibiotics, directly or indirectly through exposure to the intestinal microflora, affect local intestinal immunity. In the vast majority of cases, cytokines are short-acting mediators of local cell interactions in physiological and pathological processes in tissues (Feldmann, 2008). Cytokines are involved in autocrine, paracrine and endocrine signaling as immunomodulating agents; autocrine regulation where the cytokine acts on the cytokine-producing cell itself; paracrine, where the cytokine acts on the cell located next to the producer call; and endocrine effect, this is a remote or systemic effect when the cytokine reaches the target cell through the blood (García Morán et al., 2013). The endocrine effect is characteristic only for IL1, TNFα, IL6, MCSF in severe systemic disease, for example, sepsis (Doherty et al., 1992; Gheorghiță et al., 2015).

It has previously been shown that use of antibiotics from different groups in various animal models leads to a significant increase in the expression of genes for pro-inflammatory IFN-γ, TNF-α, IL-1β and IL-6, anti-inflammatory IL-4 and effector cytokines of the Th17 cell subpopulations IL-17 and IL-23 (Sun et al., 2019). Broad spectrum antibiotics have a relatively stronger effect on the cytokine response in the colon (Strzepa et al., 2017).

In current research the values ​​of the obtained cytokine profile in feces are local and reflect the state of the intestinal mucosa and lymphoid tissue associated with the gastrointestinal tract(Rakhim M. Khaitov, 2019). In group AB, we observe a suppression of the production of hematopoietic cytokines GCSF, Granulocyte Macrophage Colony-Stimulating Factor (GMCSF), IL3, by day 10 of the study. Under the influence of mare’s milk, we observe an increase in the production of hematopoietic cytokines GCSF, GMCSF, IL3 on day 20 of the study. It is assumed that elevated production of the latter cytokines contributes to the proliferation and differentiation of granulocytes and monocytes, and as a result, restoration of the body after a disease and increased immune system responsiveness. The obtained numbers on day 60 of the study show the normalization of the synthesis of cytokines in the studied samples produced by the intestinal lymphoid system. This is manifested by a self-regulating cascade of cytokines; in other words, in response to the production of activation cytokines, the intestinal lymphoid cells begin to produce inhibitory cytokines. In response to the activation of hematopoietic cytokines, an increase in the production of hematopoiesis inhibitors (TNFα, MIP1α) is observed. In contrast, indicators remain reduced until day 60 of the study in the AB group.

IL1 and TNFα are cytokines of primary (pre-immune) inflammation and act locally. They are necessary for the immune response mechanism to present the antigen to T-lymphocytes, promote their migration to regional lymph nodes, and create conditions for a lymphocytic immune response (Khaitov, 2019). These cytokines protect the primary link of the immune system and also include IL6 and IL8. The increase in IL1α production is balanced by the production of the endogenous anti-inflammatory agent IL1RA, which is its antagonist and limits the damaging effect of inflammatory processes on cells and tissues of the body. By the end of the study, no significant shifts were observed in the restoration of the cytokine profile in the AB group. In conclusion, it seems that this prebiotic prevents possible fluctuations in the activity of cytokines and immunoglobulins associated with the action of the antibiotic.

Our study has several limitations. Due to our strict inclusion criteria, aiming for a clinically homogeneous proband set with no previous antibiotic exposure during the three months prior to inclusion, sample size is very small, therefore the results should be interpreted with caution. Thus we conceive of this study as a hypothesis-generating pilot. However, given the stringency with which our tests were performed, for those few features where statistical significance could still be concluded, we consider those reliable, with impact of low power seen in how few such findings pass all tests. Second, we employed division of subjects into two groups, control and MM, to illustrate the influence of MM on gut microbiota. Here, bronchopneumonia and associated pathological processes equally occur in both groups, as does standard treatment (i.e. the antibiotic intervention). Thus, our findings reflect the impact of MM under circumstances of disease being treated, rather than necessarily being representative of what MM would do in healthy subjects. We do not consider this a true limitation, however, as it represents the use case for which we seek to evaluate impact of MM administration. Third, in our study there were no probands where we could investigate the effect of MM in a healthy group, and as such, we cannot distinguish microbiome changes from MM alone from those associated with microbiome restoration following antibiotics-induced disruption *via* MM. As such, our results are ambiguous with regards to whether observed patterns reflect the one or the other possibility.

Thus, further studies are needed to clarify the mechanisms of mare’s milk for local immunity. Additionally, little is known about the interaction of the microbiota, its metabolites, and the corresponding inflammatory/anti-inflammatory reactions in the intestine mediated by cytokines. Moreover, how microbiota and its metabolites affect host immunity during antibiotic therapy remains to be explored.

## Conclusion

This pilot study on the impact of mare’s milk consumption reveals it to be well tolerated in children concurrently receiving antibiotic treatment and suggests positive effects on recovery of the gut microbiome following such perturbation. Future studies with larger sample numbers will be necessary to understand in more detail the effects of mare’s milk on protecting and restoring the microbiome, as well as on the immunological response beyond the here studied age category.

## Data Availability Statement

The datasets presented in this study can be found in online repositories. The names of the repository/repositories and accession number(s) can be found below: NCBI BioProject [accession number PRJNA623574].

## Ethics Statement

This study was approved by the regional committee for medical research ethics in the National Laboratory Astana, Nazarbayev University (Protocol number 20 from 22.09.2017, IORG0006963), and registered on ClinicalTrials.gov under study identifier NCT03657836. Written informed consent to participate in this study was provided by the participants’ legal guardian/next of kin.

## Author Contributions

AK designed research. SA and KR performed patient recruitment, clinical and laboratory examination. UL analyzed data and critically revised the paper. SK, ZK, AN, MN and DB performed research. ME and LM provided key technical support and critically revised the paper. ME critically revised the paper. AK and SF wrote the paper. SF conducted computational analyses. All authors contributed to the article and approved the submitted version.

## Funding

Financial support for this study was supported from the grant AP09259975 - “Health programming, evolution of the infants’ microbiome” of the Ministry of Education and Science of the Republic of Kazakhstan.

## Conflict of Interest

Authors AK and SK were employed by the company SaumalBioTech LLP, which activity is in the area of the scientific research of mare’s milk.

The remaining authors declare that the research was conducted in the absence of any commercial or financial relationships that could be construed as a potential conflict of interest
