# Supplementary figures and images for: Dynamic Changes in Microbiome Composition Following Mare’s Milk Intake for Prevention of Collateral Antibiotic Effect
Source: Front Cell Infect Microbiol. 2021 Apr 21;11:622735. doi: 10.3389/fcimb.2021.622735 (PMC8097163; doi:10.3389/fcimb.2021.622735)

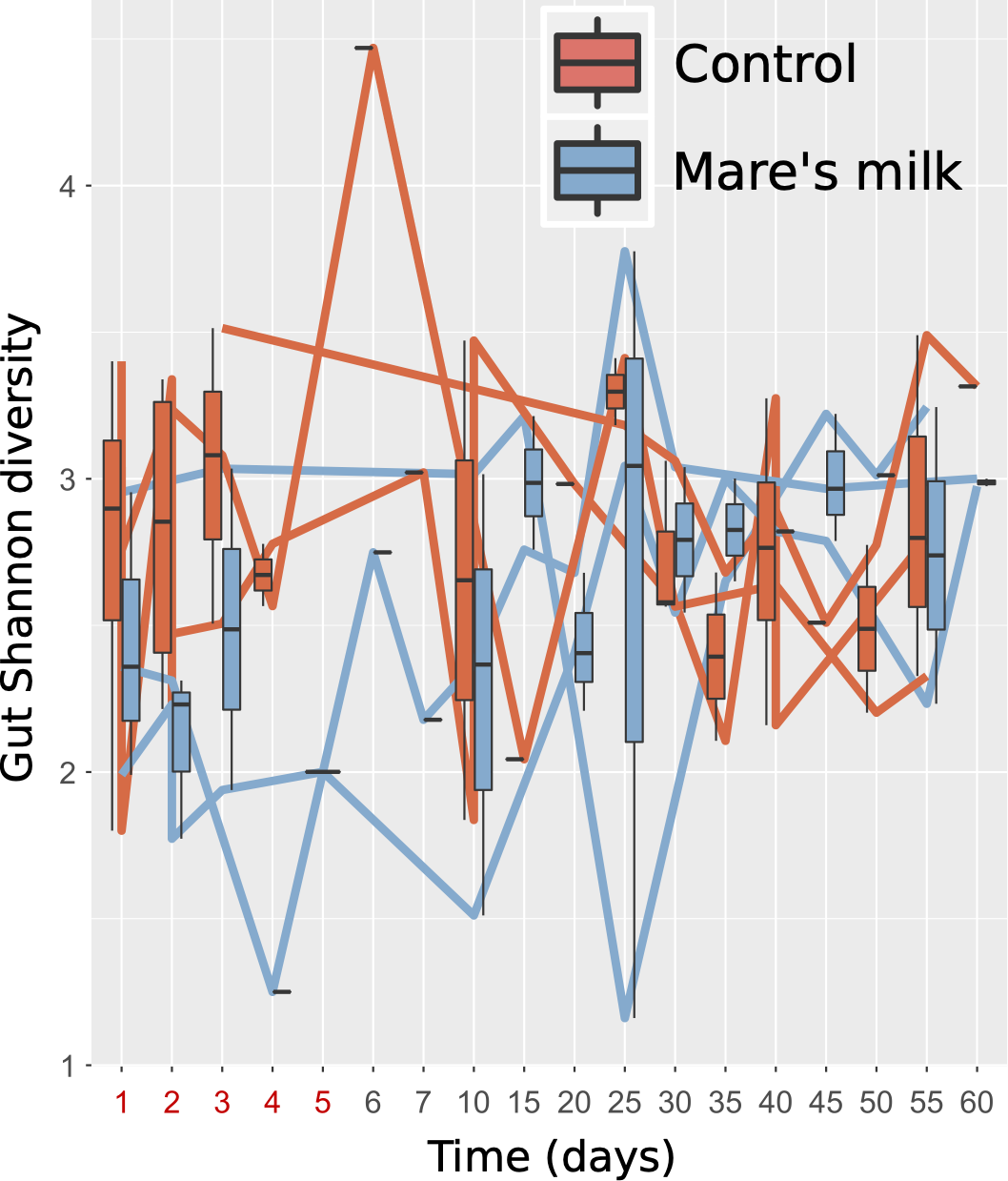

Supplement: Supplementary file 1 [file Image_1.tiff]
